# Children are Less Likely Than Adults to Develop Complete Heart Block Following TAVR

**DOI:** 10.1007/s00246-025-03889-3

**Published:** 2025-05-13

**Authors:** Claire A. Newlon, Mary C. Niu, Edem Binka, Dana M. Boucek, Zhining Ou, Susan P. Etheridge, Thomas A. Pilcher, Mary Hunt Martin, Robert G. Gray, S. Yukiko Asaki

**Affiliations:** 1 Division of Pediatric Cardiology, Department of Pediatrics, UCLA Health System, Los Angeles, CA USA; 2 Division of Pediatric Cardiology, Department of Pediatrics, University of Utah, Primary Children’s Hospital, Salt Lake City, UT USA; 3 Division of Epidemiology, Department of Internal Medicine, University of Utah, Salt Lake City, UT USA; 4 Division of Pediatric Cardiology, St. Luke’s Children’s Pediatrics, Boise, ID USA; 5 Lucile Packard Children’s Hospital Stanford, Palo Alto, CA USA

**Keywords:** TAVR, Pacemaker, Congenital heart disease, Pediatrics

## Abstract

TAVR is an alternative to surgical aortic valve [AoV] replacement. In adults, rates of atrioventricular block [AVB] requiring permanent pacemaker [PPM] placement in the modern era are 4–24%. Post-TAVR conduction abnormality incidence and risk factors are unknown in children and young adults. Describe post-TAVR conduction abnormalities in children and young adults. Retrospective single-center review of patients undergoing TAVR (9/2014 to 6/2021). Patients with pre-existing complete AVB or PPM were excluded (*N* = 1). The relationship between described adult risk factors for AVB and primary outcome of new conduction abnormality was assessed. Of 28 cases (Ages 3.5–22 y), 50% were male and 43% had isolated AoV disease, and the remainder with multilevel obstructive left-sided heart disease (29%) or complex congenital heart disease (29%). Baseline conduction abnormality was present in 57% (16/28), with right bundle branch block the most prevalent (9, 56%). Post-TAVR, acute- and late-onset conduction abnormalities occurred in 9 and 2 patients, respectively, and resolved in 8/11 patients during follow-up. One patient required PPM for complete heart block. There was no association between new conduction abnormality and previously reported adult risk factors—baseline RBBB, membranous septum length, valve implantation depth, or degree of valve oversizing. There was no relationship between outcome and baseline conduction abnormality nor history of multiple AoV interventions. In our pediatric series, AVB requiring PPM was rare following TAVR, with an incidence 3.6%—lower than average rates reported in adult literature. We identified no association of conduction abnormality with described adult risk factors.

**Supplementary Information:**

The online version contains supplementary material available at 10.1007/s00246-025-03889-3.

## Introduction

Since publication of the sentinel paper on transcatheter aortic valve replacement (TAVR) in 2010 [1], TAVR has gained increasing popularity and has become the standard of care for treatment of calcific aortic stenosis in elderly adults regardless of their surgical risk [2]. Our center has pioneered TAVR in pediatric patients with congenital heart disease (CHD). However, despite the abundance of adult literature on outcomes and complications, there are only small case series describing center-specific experiences with TAVR in the pediatric and young adult populations [3].

Conduction abnormalities, including complete heart block (CHB) requiring permanent pacemaker placement (PPM), are well-described complications of TAVR in the adult population with a variable incidence of pacemaker placement generally ranging from 4–24% in the modern era, with historical data as high as 51% [4–7]. This risk is due to the vulnerable course of the penetrating bundle of His. Based on autopsy specimens, the His bundle travels along the border of the membranous septum located inferiorly between the right and noncoronary aortic cusps, rendering it susceptible to mechanical trauma with TAVR [8]. Risk factors for CHB with the TAVR procedure—both modifiable and nonmodifiable—have been identified in the adult population. The most consistently reported risk factor for CHB is pre-existing right bundle branch block (RBBB) [4]. Other described risk factors include deep valve implantation within the left ventricular outflow tract (LVOT), prosthesis oversizing (relative to valve annulus or LVOT), shorter membranous septum, advanced age, non-coronary cusp (NCC) thickness, and the degree of NCC calcification (with the latter two potentially leading to direct compression and damage of the local conduction system) [4, 7, 9–15].

Due to the relative paucity of children and CHD patients who have undergone TAVR, conduction abnormalities after TAVR have not been described in this population. Consequently, there is a lack of data regarding risk factors in this group of patients. In fact, these patients represent a unique clinical group, exhibiting a preponderance of baseline conduction abnormalities relative to the healthy pediatric population. For this reason, some of the risk factors not directly related to advanced age, as identified in adult studies, may more accurately predict CHB within this pediatric population. Additional unknowns in pediatric TAVR patients include how peri- and post-procedural changes in ventricular loading conditions affect ectopy burden. We aim to describe our institutional outcomes concerning conduction abnormalities and ectopy burden after TAVR in pediatric and young adult patients with CHD.

## Methods

### Patient Population

All patients treated with a SAPIEN 3 valve (Edwards Lifesciences, Irvine, CA, United States) at our institution from the first case in 2014 to June 2021 were included in this study (*n* = 29). The decision to undergo TAVR was determined via a multidisciplinary discussion within our heart center involving cardiothoracic surgeons along with referring and interventional cardiologists. All patients met our institution’s clinical criteria for aortic valve replacement. All patients were provided written informed consent for the procedure. TAVR was performed via standard technique under general anesthesia using the transfemoral approach in all but *n* = 1 patients, in which a hybrid, trans-aortic approach was chosen due to the small size of the patient. To determine the incidence of conduction abnormalities after TAVR, patients with CHB and previous pacemaker were excluded. This study was approved by the local IRB.

### Prosthesis Sizing

Valve size and type were selected by the primary interventionalist and was based on careful pre-procedural measurements obtained via multi-slice computed tomography and the manufacturer’s recommendations, along with intra-procedural angiography.

### Definition of Risk Factor Variables

Risk factor variables were drawn from seminal studies on TAVR in the adult population and included: (1) pre-existing RBBB, (2) membranous septum length, (3) implantation depth, (4) membranous septum length relative to implantation depth (∆MSID) where a value less than one indicates implantation below the membranous septum, and (5) oversizing ratio—two separate variables for the ratio of the prosthesis diameter relative to (1) the aortic valve (AoV) and, (2) the LVOT diameters, respectively.

To capture risk factors which may be unique to our patient population, we included the following novel factors as part of our risk model:

1) Prior aortic valve intervention >1 (including both transcatheter and surgical), and

2) Pre-existing—or baseline—conduction abnormalities (types listed in Table 1 under baseline CA).

**Table 1:** Baseline patient characteristics

	All (*n* = 28)	Post-TAVR CA	No CA	*p*-value
Total no. patients, n	28	9	19	
Age range, years (median)	14.9 (3.5–22)	17.4 (15.1, 18.7)	14.4 (12.2, 15.6)	0.015
Female (%)	14 (50%)	4 (44.4%)	10 (52.6%)	1.000
Race, non-Hispanic white	24 (85.7%)	9 (100%)	15 (78.9%)	1.000
BSA, median (IQR)	1.6 (1.4–1.8)	1.7 (1.6, 1.8)	1.6 (1.4, 1.7)	0.099
Congenital heart disease^€^	28 (100%)	9 (100%)	19 (100%)	
Isolated aortic valve disease	12 (42.8%)	5 (55.6%)	7 (36.8%)	0.432
Multilevel obstructive left-sided disease	8 (28.6%)	2 (22.2%)	6 (31.6%)	1.000
Complex CHD	9 (32.1%)	2 (22.2%)	7 (36.8%)	0.670
Prior transcatheter or surgical AoV intervention				
Only 1 prior transcatheter	5 (17.9%)	3 (33.3%)	2 (10.5%)	0.290
Only 1 prior surgical	1 (3.6%)	0 (0%)	1 (5.3%)	1.000
Multiple AoV interventions^ƚ^	17 (60.7%)	4 (44.4%)	13 (68.4%)	0.223
Multiple transcatheter^¥^	3 (10.7%)	1 (11.1%)	2 (10.5%)	1.000
Multiple surgical	9 (32.1%)	1 (11.1%)	8 (42.1%)	0.195
Prior sternotomies > 1	13 (46.4%)	2 (22.2%)	11 (57.9%)	0.148
Baseline coronary anomalies	4 (14.3%)	1 (11.1%)	3 (15.8%)	1.000
History of coronary reimplantation	1 (5%)	0 (0%)	1 (5.3%)	1.000
Anomalous origin	2 (7.1%)	0 (0%)	2 (10.5%)	1.000
Dilated coronary	1 (5.3%)	1 (11.1%)	0 (0%)	0.321
Comorbidities				
Genetic syndrome	2 (7.1%)	1 (11.1%)	1 (5.3%)	1.000
Liver dysfunction	1 (5.3%)	0 (0%)	1 (5.3%)	1.000
Renal dysfunction	1 (5.3%)	0 (0%)	1 (5.3%)	1.000
Neurological disorder	4 (14.3%)	0 (0%)	4 (21.1%)	0.273
Patients with baseline CA*	16 (57.1%)			
Types of baseline CA (n = 16)	20			
PR prolongation	4 (20%)			
NIVCD	5 (25%)			
Incomplete RBBB	4 (20%)			
RBBB	5 (25%)			
Incomplete LBBB	1 (5%)			
LBBB	1 (5%)			

Values expressed as median (IQR) or *n* (%)

^€^One patient qualified as both multilevel obstructive left-sided disease and complex CHD

^ƚ^Either transcatheter or surgical AoV interventions

^¥^Excluding surgical AoV interventions

^¶^Excluding transcatheter AoV interventions

^*^Some patients exhibited multiple levels of conduction abnormalities

*CA* Conduction Abnormalities, *BSA* Body Surface Area, *AoV* Aortic Valve, *CHD* Congenital Heart Disease

Procedure-specific data were also collected but were not included as primary risk factors in the forest plot. These variables included the incidence of valve-in-valve procedures, whether the surgical bioprosthetic valve frame was intentionally fractured prior to valve implantation, the final valve diameter at time of implantation, and the presence of device malposition during the case.

### Multislice Computed Tomography Data Analysis

Multi-slice computed tomography (MSCT) datasets from patients were transmitted to a dedicated workstation for multiplanar reconstructions. The membranous septum length was measured from the aortic annular plane to the most superior portion of the muscular interventricular septum in the coronal view as described in prior studies [9, 16]. From this view, the AoV annular diameter and the LVOT diameter (within 2–5 mm of the aortic valve annular plane) were measured. Oversizing ratio was calculated as a ratio of prosthesis diameter to both the LVOT and the aortic valve annular diameters, respectively, as measured on MSCT images. No correction was performed for eccentricity.

### Determination of Implantation Depth

The implantation depth was determined via angiographic data measured offline using Siemens Syngo Dynamics Workplace (Siemen Healthcare GmbH, Siemens Medical Solutions USA Inc.). The measurements were obtained, as described in prior studies [17], as the distance from base of the noncoronary cusp to the prosthesis stent inflow from aortic root injection post-TAVR final deployment. Angiographic data were missing for two patients. Implantation depth was reported both as an absolute measurement (mm) and as a ratio of this angiographic measurement (i.e., valve projection into the LVOT) to the membranous septum length as measured on pre-procedure MSCT (see prior section).

### Electrocardiographic and Arrhythmia Analysis

Twelve or 15-lead electrocardiograms (ECGs) were recorded on admission, immediately post-procedure, before discharge, and at follow-up. These were reviewed by 2 physicians (CN, SYA) according to recommendations issued by the 2009 American Heart Association/American College of Cardiology Foundation, and Heart Rhythm Society [18]. Patients’ medical records were reviewed for documentation of arrhythmias while inpatient, as telemetry data is not stored after discharge.

### Echocardiographic Analysis

Echocardiograms performed at three time-points: (1) pre-procedure, (2) during post-procedure admission, and (3) at follow-up visit (1–5 months) were reviewed. Patient-specific, pre-procedural, and intra-procedural parameters were collected, including left ventricular (LV) dysfunction, LV end diastolic dimension and Z-score, presence of LV hypertrophy, aortic valve peak and mean gradients, as well as the degree of valvar and paravalvar insufficiency.

### Statistical Analysis

Demographics and clinical outcomes of interest were summarized using mean and standard deviation (SD) or median and interquartile range (IQR) for continuous variables. Counts and percentages were reported for categorical variables. Given the small sample size, hypothesis testing was not conducted. Instead, 83.7% confidence intervals (CIs) were generated for continuous variables with Wilson score-based method [19]. Binary variables were represented by their proportion of events and 83.7% CI of the proportion. Two groups were statistically non-significant (*p*-value > 0.05) if their corresponding 83.7% CI intervals overlap. Statistical significance was assessed at the 0.05 level. Statistical analyses were implemented using R v. 4.0.3 [20].

## Results

### Patient Demographics

Over the study period, 29 patients underwent TAVR—using the Edwards Sapien 3 (Edwards Lifesciences, Irvine, CA, USA) balloon-expandable valve. One patient with pre-existing AVB and PPM was excluded. In the study population (age range 3.5–22 years [median 14.9 years], 50% male), 42.9% had isolated AoV disease, and the remaining had left-sided cardiac lesions in-series, or complex CHD. The majority of patients had undergone at least one prior transcatheter (*n* = 18, 64%) or surgical (*n* = 15, 53.6%]) AoV intervention. A minority of patients had baseline coronary anomalies (*n* = 4, 14.3%), but without evidence of coronary stenosis. Two patients (7.1%) had genetic syndromes: Mosaic Turner syndrome and 22q11.2 deletion syndrome. Patients with new-onset post-TAVR conduction abnormalities were older (median 17.4 vs. 14.4 years, *p* = 0.015) but otherwise there were no statistical differences in pre-procedural characteristics between patients with and without post-TAVR conduction abnormality. Comprehensive patient demographics are listed in Table 1.

### Conduction Characteristics Pre- and Post-TAVR

At baseline, 16 (57.1%) patients demonstrated conduction abnormalities (Table 1). Conduction abnormalities included 1st degree AVB (*n* = 4, 20%), nonspecific intraventricular conduction delay (NIVCD, *n* = 5, 25%), incomplete RBBB (iRBBB, *n* = 4, 20%), RBBB (*n* = 5, 25%), incomplete LBBB (iLBBB, *n* = 1, 5%), and LBBB (*n* = 1, 5%). Note that four patients had multilevel abnormalities consisting of 1st degree AVB in addition to an intraventricular delay vs. bundle branch block (see Table 1).

Immediately following TAVR, 9/28 (32.1%) patients had new-onset conduction abnormalities, two of whom (22.2%) had multiple levels of conduction abnormalities (Table 2). First degree AVB (*n* = 4) and LBBB (*n* = 6) were the most frequent acquired conduction abnormalities, with one instance each of 2nd and 3rd degree AVB. Two patients had late-onset LBBB which was first noted one-week post-TAVR.

**Table 2:** Post-TAVR conduction abnormalities (CA)

Patients with CA post-TAVR	9 (32.1%)
Types of post-TAVR CA*	
PR prolongation	4 (33.3%)
2nd degree, type 2 AVB	1 (8.3%)
3rd degree AVB	1 (8.3%)
LBBB	6 (50.0%)
Patients with multilevel CA	2 (22.2%)
PR prolongation and LBBB (n = 1)	
PR prolongation, 2nd degree, type 2 AVB, and LBBB (n = 1)	
Patients with late-onset CA (> 1 week post-TAVR) LBBB	2 (22.2%)
Patients without follow-up at our institution	2 (22.2%)
Resolution of CA^ƚ^	
PR prolongation	3/3 (100%)
2nd degree, type 2 AVB^⁋^	1/1 (100%)
3rd degree AVB^¥^	0/1 (0%)
LBBB	3/5 (60.0%)

Values expressed as *n* (%)

*NIVCD* Non-Specific Intraventricular Conduction delay; *RBBB* Right Bundle Branch Block, *LBBB* Left Bundle Branch Block, *AVB* Atrioventricular Block

^*^Some patients exhibited multiple levels of conduction abnormalities

^ƚ^Two patients had late-onset LBBB at initial follow-up post-TAVR with resolution within 1 month, *n* = 1, and *n* = 1 was lost to follow-up

^⁋^Resolution on Holter completed at 1 month follow-up

^¥^Patient undergoing valve-in-valve procedure (prior valve: Sapien XT), presence of baseline LBBB (QRS 160 ms), prior sternotomies: 3, prior transcatheter AoV interventions: 6, history of two prior mechanical MVR

Among patients with follow-up data available (*n* = 26), 100% of 1st and 2nd degree AVB, and 60% of LBBB, had resolved by one-year post-procedure. Persistent conduction abnormalities were seen in 3 patients and included LBBB (*n* = 2) and 3rd degree AVB requiring PPM the following day (*n* = 1, 3.6% total cases). The latter patient had a valve-in-valve procedure which included overexpansion of their prior Sapien XT, in addition to baseline LBBB (QRS 160 ms) with a history of 3 prior sternotomies, 6 transcatheter AoV interventions, 2 prior mechanical mitral valve replacements.

Of the 4 patients with multiple conduction abnormalities at baseline, only one patient experienced new post-TAVR conduction abnormality—1st degree AVB—which then resolved at follow-up.

### Risk Factors for Conduction Abnormalities Post-TAVR

**Fig. 1:**
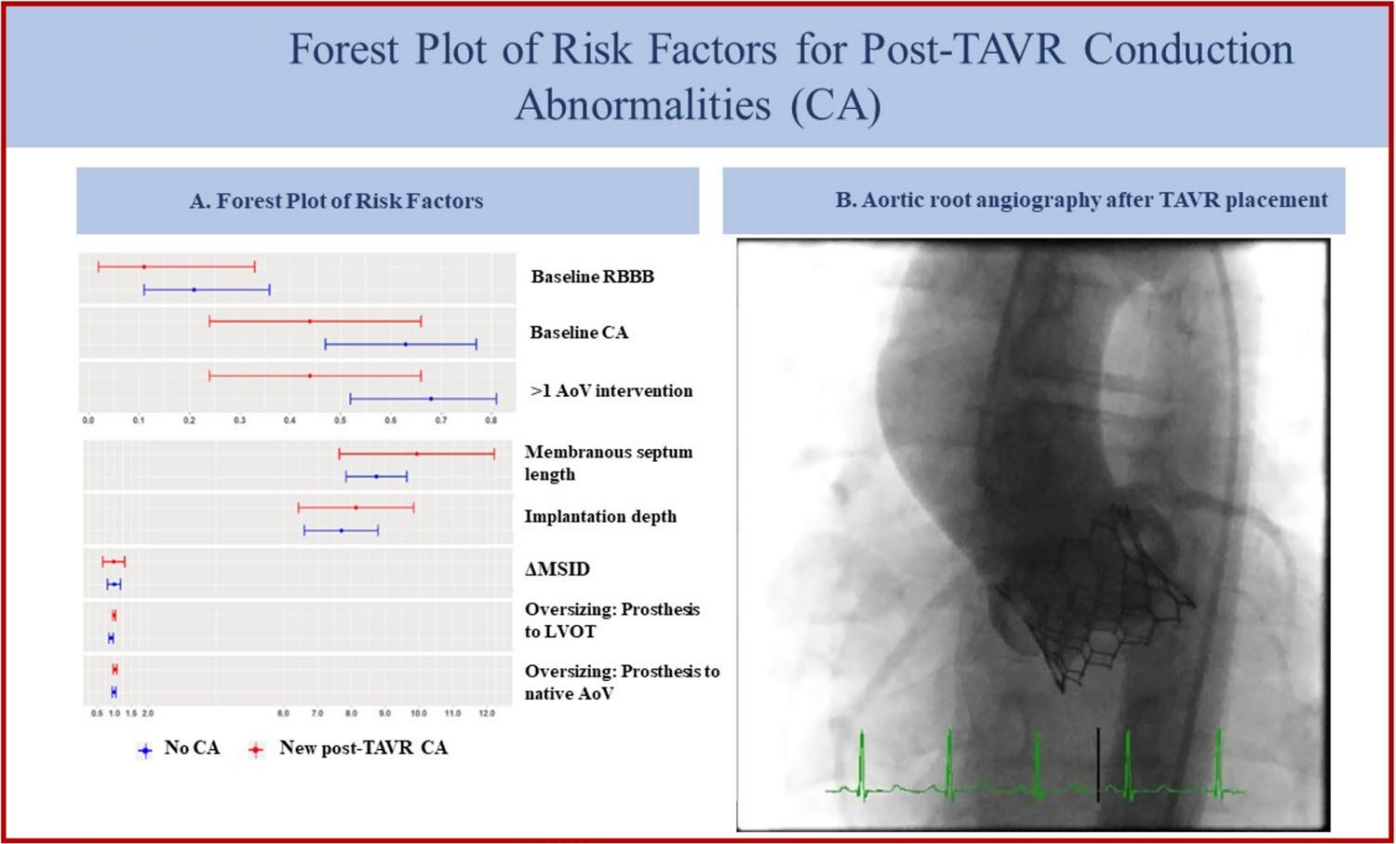
Forest plot of risk factors for post-TAVR conduction abnormalities (CA)

Due to the small study population, we used a forest plot to assess for the effect of risk factors on the primary outcome of any new conduction abnormalities including PPM. There was no significant association between post-TAVR conduction abnormality incidence and the risk factors reported in the adult literature, including presence of baseline RBBB, membranous septum length, valve implantation depth, or degree of valve oversizing– relative to both valve annulus and LVOT. Additionally, there was no relationship between outcome and any degree of baseline conduction abnormality or history of > 1 prior surgical or transcatheter AoV intervention. See Fig. 1 and Table 3.

**Table 3:** Risk factors for conduction abnormalities (CA)

	Post-TAVR CA (*n* = 9)	No CA (*n* = 19)	*p*-value
Baseline Characteristics			
Baseline RBBB, n	1 (11.1%)	4 (21.1%)	1.000
Pre-TAVR AoV diameter^†^, mm	26.1 (3.2)	24.7 (5.0)	0.316
Pre-TAVR LVOT diameter^†^, mm	27.6 (3.1)	27.3 (6.5)	0.457
Intra-Procedural Characteristics, n			
ViV	1 (11.1%)	8 (42.1%)	0.195
Intentional BPV frame fracture	0 (0%)	4 (21.1%)	0.338
Sapien 3 Valve size			–
20 mm	0 (0%)	4 (21.1%)	0.195
23 mm	2 (22.2%)	8 (42.1%)	–
26 mm	2 (22.2%)	3 (15.8%)	–
29 mm	5 (55.6%)	4 (21.1%)	–
Device Malposition	0 (0%)	0 (0%)	N/A
Device Positioning Characteristics			
Membranous septum length^†^, mm	9.9 (4.5)	8.7 (2.7)	0.664
Implantation depth*	8.1 (3.3)	7.7 (3.1)	0.782
ΔMSID	1.1 (0.6)	1.1 (0.6)	0.843
Degree of valve oversizing			
Prosthesis d./LVOT d.^†^	1.0 (0.1)	0.9 (0.2)	0.211
Prosthesis d./AoV annulus d.^†^	1.0 (0.1)	1.0 (0.2)	0.455

Values expressed as mean (SD) or *n* (%)

^†^CTA

^*^Angiography

*AoV* Aortic Valve, *LVOT* Left Ventricular Outflow Tract, *ViV* Valve-In-Valve, *BPV* Bioprosthetic Valve, *ΔMSID* Ratio of Membranous Septum Length to the Implantation Depth, a value < 1 indicates implantation below the membranous septum; *d* diameter

There was no measurable difference between groups with and without post-TAVR conduction abnormalities in terms of native vs valve-in-valve procedures, intentional bioprosthetic valve frame fracture, or the absolute size to which the valve was dilated. There were no incidences of valve malposition or embolization Table 3.

### Echocardiographic Data

Prior to TAVR, 3 (10.7%) patients demonstrated LV dysfunction (EF < 55%) on echocardiogram and more than half of subjects (*n* = 16, 57.1%) had LV hypertrophy. At follow-up several months after TAVR (median 98 days, IQR 28.5, 139.5 days), LV dysfunction had resolved in all patients, LV hypertrophy had resolved in 50% (*n* = 8), and the aortic valve mean gradient improved from a median of 42 (IQR 34.5, 56.5) to 16.5 mmHg (IQR 13.8, 24.0). No patients had significant AI and only one patient had greater than mild paravalvar leak (Table 4).

**Table 4:** Echocardiographic data before and after TAVR

	Pre-TAVR (*n* = 28)	Immediately Post-TAVR (*n* = 28)	Follow-up (*n* = 27)
Days before/after procedure	66.0 (105.8, 31.5)	1 (1.0, 1.0)	98 (28.5, 139.5)
LV dysfunction (EF < 55%)	3 (10.7%)	3 (10.7%)	0 (0%)
40–55%	1 (3.6%)	2 (7.1%)	0 (0%)
30–39%	1 (3.6%)	1 (3.6%)	0 (0%)
< 30%	1 (3.6%)	0 (0%)	0 (0%)
LV size (LVIDd, cm)	5.2 (4.7, 5.7)	4.8 (4.3, 5.3)	4.9 (4.3, 5.4)
LV size, indexed*	1.4 (−0.1, 2.4)	0.1 (−0.8, 1.7)	0.2 (−1.1, 1.2)
LV hypertrophy (Y)	16 (57.1%)	12 (42.9%)	8 (29.6%)
If yes, LV mass Z-score	4.4 (3.6, 5.1)	3.2 (2.8, 4.4)	4.2 (2.6, 5.3)
AoV stenosis (Y)	24 (85.7%)	23 (82.1%)	25 (92.6%)
AoV mean gradient	42.0 (34.5, 56.5)	15.5 (12.0, 23.0)	16.5 (13.8, 24.0)
AoV peak gradient	71.5 (58.5, 100.0)	26.0 (21.2, 35.0)	25.5 (20.0, 34.5)
AoV insufficiency			
None	2 (7.1%)	21 (75%)	18 (66.7%)
Trace	1 (3.6%)	7 (25%)	8 (29.6%)
Mild	5 (17.9%)	0 (0%)	1 (3.7%)
Mild to moderate	7 (25%)	0 (0%)	0 (0%)
Moderate	8 (28.6%)	0 (0%)	0 (0%)
Moderate to severe	4 (14.3%)	0 (0%)	0 (0%)
Severe	1 (3.6%)	0 (0%)	0 (0%)
Paravalvar leak			
None	25 (89.3%)	23 (82.1%)	22 (81.5%)
Trace	0 (0%)	1 (3.6%)	2 (7.4%)
Mild	1 (3.6%)	3 (10.7%)	2 (7.4%)
Mild to moderate	1 (3.6%)	1 (3.6%)	0 (0%)
Moderate	0 (0%)	0 (0%)	1 (3.7%)

Values expressed as median (IQR) or *n* (%)

^*^LVIDd Z-score

*LV* Left Ventricle, *EF* Ejection Fraction, *Y* Yes or Present, *AoV* Aortic Valve

### Arrhythmias and Ectopy Pre- and Post-TAVR

Three patients had documented arrhythmias prior to TAVR including one with atrial ectopic tachycardia (AET), a second individual with intra-atrial reentrant tachycardia and atrial fibrillation (AF), and a third subject with history of intermittent accelerated ventricular rhythm (AVR). The patient with AVR was treated with low dose nadolol prior to TAVR, but other patients were not started on antiarrhythmics prior to TAVR.

Following TAVR, no patients experienced clinically significant arrhythmias including no sustained, hemodynamically significant, or frequent arrhythmias. Transient post-procedure arrhythmias were seen in 17.8% (*n* = 5) of patients including AVR or nonsustained ventricular tachycardia (*n* = 3, 10.7%), and AET (*n* = 2, 7.1%). Holter monitors were performed in 39.3% (*n* = 11) of patients. No new conduction abnormalities were identified on post-discharge ambulatory ECG monitoring.

### Short to Mid-Term Outcomes

At follow-up (median 98 days), up to one-year, there were no new or progressive conduction abnormalities, reinterventions, or deaths.

## Discussion

In our pediatric and young adult population with CHD undergoing TAVR, CHB and the need for PPM was rare, with an incidence (3.6%), lower than that reported in adult patients (4–24%) [5, 13, 21, 22]. Less advanced conduction abnormalities sustained following TAVR largely resolved within a year. Selected risk factors well-described in adult patients [9, 14, 23, 24], including baseline presence of RBBB, membranous septum length, implantation depth, and oversizing ratio, were not significantly associated with PPM (*n* = 1) or new conduction abnormalities (*n* = 9) in our population. Additionally, pre-procedural risks including any degree of baseline conduction abnormalities and > 1 AoV interventions, were not associated with new post-TAVR conduction abnormality. To our knowledge, this is the first study in pediatric and young adult patients, and in those with complex CHD, assessing electrical consequences following TAVR.

To date, studies investigating predictors for post-TAVR PPM have exclusively involved adult populations. TAVR patients have a mean age of 70–80 years, with high rates of baseline subaortic and leaflet calcification, and thus represent a distinctly different patient population than children and young adults with CHD [4, 13, 22]. Studies focusing on coronary artery calcium deposition in the “young” (i.e., ≤ 45 years old) adult population have excluded those with CHD [25]. Furthermore, the molecular mechanisms of valve disease may be different—i.e., excessive extracellular matrix deposition without calcification—between adult and pediatric aortic valve stenosis [26]. This has led to TAVR risk-stratification systems specific to the unaltered aging process, including the “calcification score” which quantifies valvar calcium deposition [27].

The existing data creates a map of risk factors for post-TAVR PPM in which procedure-specific, cardiac-specific, and aging-specific factors are interwoven and inextricable—highlighting the need for focused research in the pediatric and young adult population. We lack appropriate pediatric risk models for post-TAVR CHB. This study establishes a baseline for this specific patient population and offers an initial reference point to create a foundational framework for developing risk models.

Longer-term prediction tools with improved accuracy and applicability will become increasingly relevant for the pediatric cardiologist when counseling younger patients and their families regarding the various treatment options for aortic valve disease. It has been almost a decade since the FDA approved the TAVR procedure, initially restricted to patients with high surgical risk. Although use has expanded to patients < 65 years old with low surgical risk, its use remains relatively rare in the pediatric population. We are beyond the need for studies of procedural efficacy and must pivot to the long-term effects of the TAVR procedure, including valvar longevity and the morbidity and mortality associated with eventual explantation [28]. Patients undergoing TAVR at a young age anticipate decades of survival, highlighting the importance of longitudinal data. Therefore, long-term safety and efficacy data—especially in these evolving young adult populations—could be practice-changing.

The pediatric cardiac conduction system differs from that of older adults in several ways, including faster conduction velocity, shorter refractory periods, and less fibrosis and sclerosis. Acquired injury to the conduction system from ischemia or invasion of aortic valve calcification into the His bundle and infrahisian conduction system are more common in adults. Even in those who did experience post-TAVR conduction abnormalities in our series, the changes were almost always transient. Additionally, the younger patients were less likely to have new conduction abnormalities, which may be secondary to the health of the conduction system or the compliance and adaptability of the LVOT in younger children. No patients experienced progressive conduction abnormality at final follow-up visit.

In addition to identifying risk factors, the literature in adult patients aims to dictate who should receive an ambulatory ECG monitor at the time of discharge. However, in our population, none of the remote monitors (*n* = 11) demonstrated new evidence of abnormal conduction, despite a skew towards placement of a monitor on patients who experienced immediate post-procedural arrhythmias. Additionally, the benign, transient nature of the arrhythmias that *were* identified suggest that there may be no routine indication for monitors in this younger population. Future research will help to define these practice recommendations.

## Limitations

This study was limited by the small population size (*n* < 30), which prohibited performing a traditional regression analysis of risk factor modification of PPM and conduction abnormality incidence.

Data accuracy for some variables was limited by the retrospective study design. Intra-procedural documentation typically does not contain granular data regarding periprocedural issues with conduction abnormalities or arrhythmias unless they were persistent and significant. Unreported brief abnormalities likely have no clinical significance. Ultimately, detailed evaluation of the periprocedural, dynamic effect of TAVR placement on conduction, as described in more recent papers [29, 30], was not plausible and warrants further study in this population. Likewise, measurement of implantation depth on angiography was challenging in this pediatric cohort, where the priority is often given to limiting contrast exposure making it challenging to discern the border of the NCC for measurements.

Finally, this was a retrospective study with limited follow-up. By necessity, patients requiring TAVR in childhood or young adulthood will be committed to a greater number of life-time interventions than an elderly patient. The presence of CHD and prior surgical palliation likely predisposes patients to earlier-onset, and increased incidence, of calcific valve disease, potentially further shortening the lifespan of a bioprosthetic valves [31]. It is unknown how earlier TAVR will impact TAVR versus surgical valve candidacy in late adulthood, when adult risk factors coalesce with risks secondary to chronic heart disease. Further longitudinal studies are needed to fully elucidate the long-term outcomes in this population.

## Conclusion

Permanent heart block and PPM placement were rare in a population of children and young adults undergoing TAVR for treatment of CHD. Presence of baseline RBBB, membranous septum length, implantation depth, oversizing ratio, baseline conduction abnormalities, and > 1 prior aortic valve intervention were not associated with new post-TAVR conduction abnormality.

## Supplementary Information

Below is the link to the electronic supplementary material.

Supplementary file1 (DOCX 14 KB)

## Data Availability

No datasets were generated or analysed during the current study.
