# Supplementary material for: Children are Less Likely Than Adults to Develop Complete Heart Block Following TAVR
Source: Pediatr Cardiol. 2025 May 13;47(3):1169–77. doi: 10.1007/s00246-025-03889-3 (PMC12901118; doi:10.1007/s00246-025-03889-3)
Supplement: Supplementary file 1 — Supplementary file1 (DOCX 14 KB) [file 246_2025_3889_MOESM1_ESM.docx]

**SUPPLEMENTAL DATA**

| **Table S1. Summary of Ectopy and Tachyarrhythmias.** | | |
| --- | --- | --- |
| **Variable** | | **Total (n=28)** |
| Presence of Baseline Arrhythmia | | 3 (10.7%) |
|  | IART and AF | 1 (3.6%) |
|  | AET | 1 (3.6%) |
|  | Accelerated ventricular rhythm | 1 (3.6%) |
| Baseline Anti-arrhythmic Use | | 1 (3.6%) |
| Intra-procedural Arrhythmia* | | 1 (3.6%) |
| New Arrhythmia Post-TAVR | | 7 (25%) |
|  | AET^ƚ^ | 2 (7.1%) |
|  | Isolated PVCs | 4 (14.3%) |
|  | NSVT or AIVR^ƚ^ | 3 (10.7%) |
|  | VT^¥^ | 2 (7.1% |
|  | Accelerated Junctional | 1 (3.6%) |
| Ambulatory ECG Monitor Post-TAVR^¶^ | | 11 (39.3%) |
| *Accelerated junctional rhythm  ^ƚ^Only identified on monitor performed after discharge  ^¥^Both cases occurred during inpatient admission; one patient with a 10-beat episode during a 2-week admission, the second patient with a single 6-beat run which did not recur on Holter monitor placed at time of discharge.  ^¶^Within 3-6 months of TAVR; included Holter and Zio Patch.  IART = intra-atrial re-entrant tachycardia; AF = atrial fibrillation; AET = ectopic atrial tachycardia; cath = cardiac catheterization; NSVT = non-sustained ventricular tachycardia; AIVR = accelerated idioventricular rhythm; VT = ventricular tachycardia; PVC = premature ventricular contraction. | | |
|  |  |  |
|  |  |  |
